# Supplementary material for: Tillage Promotes the Migration and Coexistence of Bacteria Communities from an Agro-Pastoral Ecotone of Tibet
Source: Microorganisms. 2022 Jun 13;10(6):1206. doi: 10.3390/microorganisms10061206 (PMC9231252; doi:10.3390/microorganisms10061206)
Supplement: Supplementary file 1 [file microorganisms-10-01206-s001.zip › microorganisms-1757234-supplementary.pdf]

# Tillage promotes the migration and coexistence of bacteria communities in an agro-pastoral ecotone of Tibet, China

Yuhong Zhao [a,1], Mingtao Wang [a,1], Yuyi Yang [b,c], Peng Shang [a], Weihong Zhang [b,c*]

[a] Tibet Agricultural and Animal Husbandry University, Nyingchi, 860000, China

[b] Key Laboratory of Aquatic Botany and Watershed Ecology, Wuhan Botanical Garden, Chinese Academy of Sciences, Wuhan, 430074, China

[c] University of Chinese Academy of Sciences, Beijing 100049, China

[1] The authors make equal contributions to this manuscript.

Corresponding author

Dr. Weihong Zhang (zhangweihong@wbgcas.cn)

Address: Wuhan Botanical Garden, Lumo Road No.1, Wuchang District, Wuhan, P.R. China. Phone: +86-27-87700853; Fax: +86-27-87510251.

**Table S1.** The alpha diversity indexes of the bacterial community in cropland and grassland.

| | | Chao1 | Shannon | Goods coverage | Simpson | Pielou evenness index | Observed species |
|---|---|---|---|---|---|---|---|
| Cropland | WL1 | 3918.4 | 11.0 | 0.9892 | 0.9991 | 0.9222 | 3841.6 |
| | WL2 | 4307.0 | 11.0 | 0.9815 | 0.9991 | 0.9199 | 4042.5 |
| | WL3 | 3487.2 | 10.8 | 0.9886 | 0.9989 | 0.9195 | 3352.2 |
| | CP1 | 3341.3 | 10.5 | 0.9862 | 0.9987 | 0.9036 | 3139.0 |
| | CP2 | 4009.3 | 10.8 | 0.9820 | 0.9989 | 0.9104 | 3706.6 |
| | CP3 | 4272.0 | 10.9 | 0.9799 | 0.9990 | 0.9125 | 3891.8 |
| Grassland | NG1 | 4496.8 | 10.9 | 0.9799 | 0.9988 | 0.9059 | 4251.9 |
| | NG2 | 3802.3 | 10.7 | 0.9878 | 0.9986 | 0.9012 | 3703.3 |
| | NG3 | 4212.9 | 10.8 | 0.9831 | 0.9986 | 0.8987 | 4043.9 |
| | EG1 | 3873.9 | 10.9 | 0.9857 | 0.9989 | 0.9192 | 3692.6 |
| | EG2 | 4894.9 | 11.2 | 0.9768 | 0.9991 | 0.9228 | 4457.4 |
| | EG3 | 4227.6 | 11.1 | 0.9837 | 0.9991 | 0.9269 | 3955.5 |
| *p*-value | | 0.15 | 0.39 | 0.46 | 0.49 | 0.70 | 0.09 |

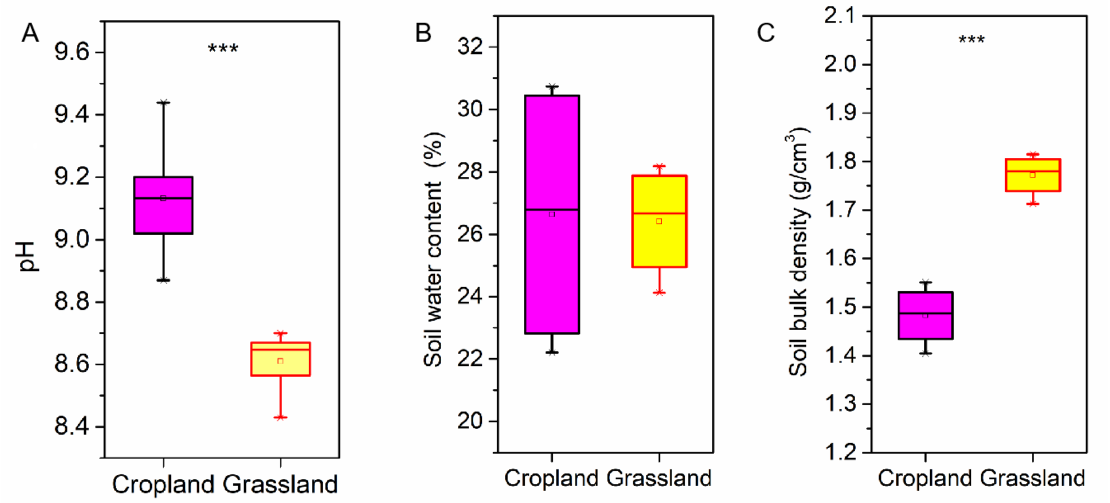


**Figure S1**. The difference of soil physicochemical properties between cropland and grassland via independent-samples T-test. *** showed a significant difference at the 0.001 level.

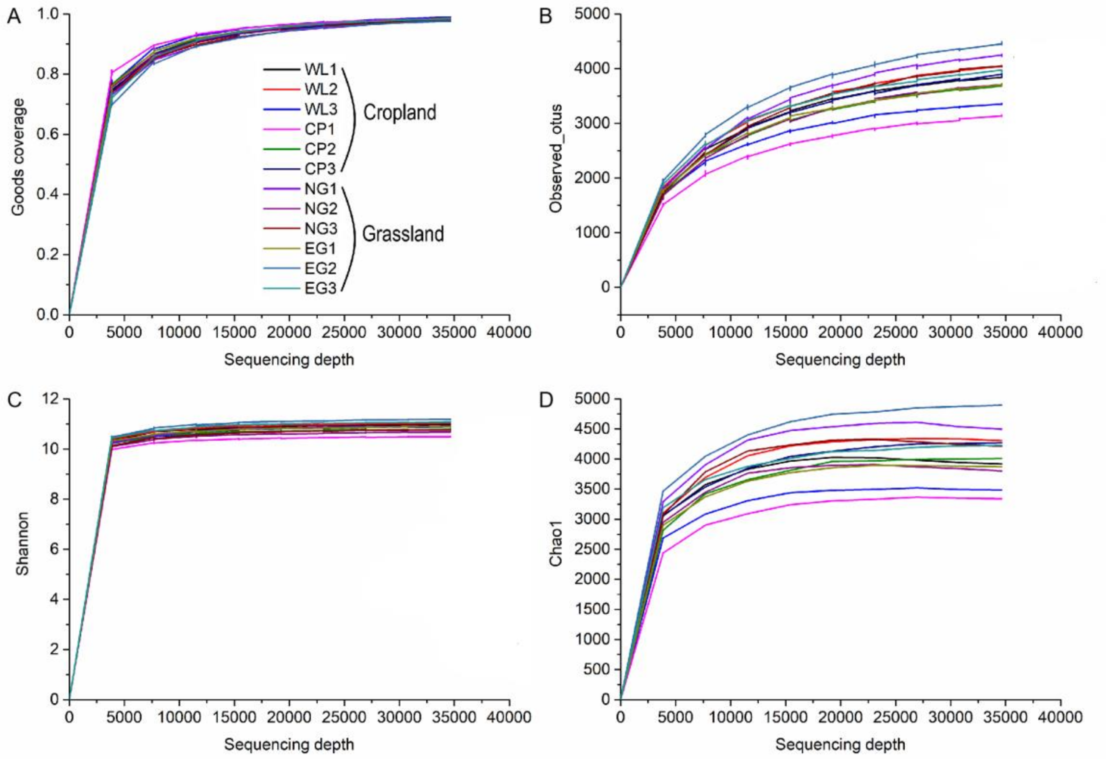


**Figure S2**. The rarefaction curves of Good's coverage (Fig. S2A), observed OTUs number (Fig. S2B), Shannon (Fig. S2C), and Chao1 (Fig. S2D) of the bacterial communities in cropland and grassland.

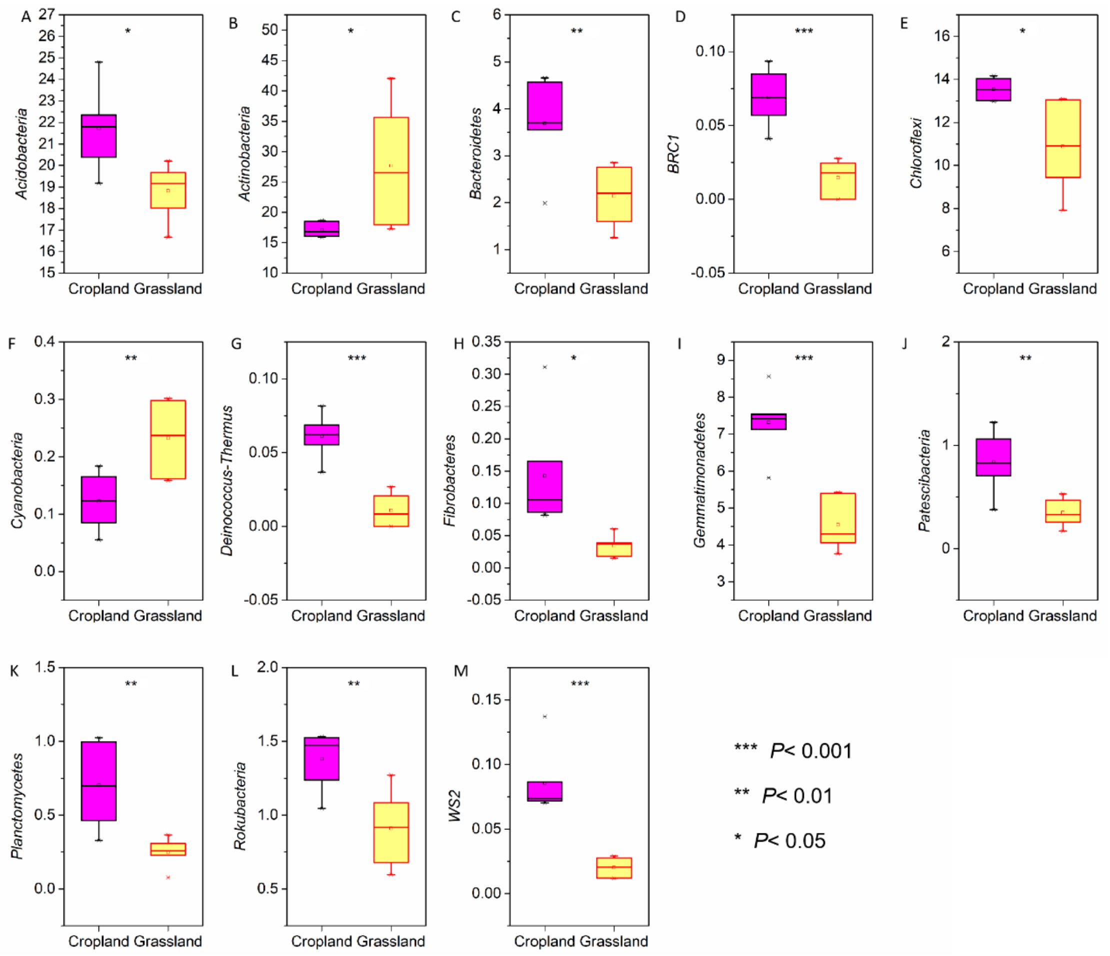


**Figure S3**. The difference analysis of bacterial communities between the cropland and grassland at the phylum level via independent sample t-test. The *** was a significant difference in the bacteria between cropland and grassland at the $p < 0.001$ level, the ** was a significant difference at the $p < 0.01$ level, and the * was a significant difference at the $p < 0.05$ level.
